# Validation of the Fitbit Zip for monitoring physical activity among free-living adolescents

**DOI:** 10.1186/s13104-016-2253-6

**Published:** 2016-09-21

**Authors:** Margaret Schneider, Larissa Chau

**Affiliations:** Social Ecology I, University of California, Irvine, Irvine, CA 92697 USA

**Keywords:** Activity monitor, ActiGraph, Adolescent, Validation

## Abstract

**Background:**

The widespread availability of affordable consumer-oriented devices for monitoring physical activity offers an appealing option to physical activity researchers, but studies are needed to demonstrate the validity and reliability of these products. To examine the validity of the Fitbit Zip, we recruited three cohorts (N’s = 25, 35, and 27) of middle-school students to wear the Fitbit and the ActiGraph simultaneously for a week. Adolescents were healthy volunteers representing a range of activity levels. Mean daily minutes of MVPA and mean steps per day were compared between the Fitbit Zip and the Actigraph.

**Results:**

The step data for the Fitbit Zip correlated highly with the step data yielded by the ActiGraph (r’s = .72, .92, .96), and the MVPA data for the Fitbit Zip correlated highly with the MVPA data from the ActiGraph (r’s = .67, .79, .94). Bland–Altman plots revealed that the Fitbit Zip overestimated activity in comparison to the ActiGraph, especially for Cohort One, which completed the study before Fitbit modified their algorithms to count as activity only bouts that continued for at least 10 min.

**Conclusions:**

Our data suggest that the Fitbit Zip is a reasonable alternative to the ActiGraph for estimating activity among free-living adolescents. However, data from the Fitbit should not be used interchangeably with data from the ActiGraph, as there is a consistent tendency for the Fitbit to overestimate steps in comparison to the ActiGraph. Also, the findings confirm concern about using for research a consumer-oriented device that does not make public their algorithms.

## Background

The past decade has seen an exponential increase in the availability of affordable, consumer-oriented devices for monitoring physical activity. The relatively low prices and attractive design of these products make them appealing to researchers who incorporate physical activity monitoring into their studies, since these features make it possible to utilize the monitors on large samples and improve participants’ compliance with wearing the device, but data on device validity is needed to support using these consumer-oriented activity monitors in a scientific context. One of the most successful companies competing in this area is Fitbit, which offers a line of products that vary in terms of how they are worn (e.g., clipped onto clothing or worn on a wrist) as well as in terms of their features (e.g., rechargeable versus battery-powered; different types of visual displays). In 2015, Fitbit held 27 % of the market share in wearable devices, ahead of Apple at 15 % [1].

The Fitbit Zip is one consumer-oriented device that has several features that make it attractive to researchers. Firstly, it is relatively affordable, (60 U.S. Dollars each), thus making it amenable to use with large samples. Secondly, it syncs wirelessly through Bluetooth, thus making transfer of data very easy. Thirdly, it runs on a replaceable battery that is projected to support continuous function for up to 6 months, meaning that the device does not have to be recharged every few days. Finally, it is small, comes in a variety of colors, and can be worn on the waist, in a pocket, or attached to undergarments; all features that make it more acceptable to users and therefore increase compliance from research participants. All of these features make the Fitbit Zip very appealing to researchers who wish to document physical activity among free-living individuals over longer periods of time.

The Fitbit Zip has been validated within a laboratory situation and has been found to be quite accurate in detecting number of steps when an adult is walking on a treadmill [2, 3], engaging in a variety of activities in a supervised setting [4], or participating in free-living activities [3]. More relevant to field-based studies, a high correlation (r = .91) has been found between daily step counts recorded by the Fitbit and the ActiGraph GT3X (considered a highly accurate research device for monitoring activity) among 47 free-living adults who wore the device for 7 days [5]. Similarly, a study of 21 adults who wore the Fitbit Zip and the ActiGraph for 48 h confirmed the high correlation between the Fitbit Zip and the ActiGraph for step counts and also showed a high correlation (r = .88) between the measures of moderate-to-vigorous physical activity yielded by each device [6]. All of the studies cited above were conducted among healthy adults.

These field-based studies go a long way toward building confidence in the Fitbit Zip as a device that can be expected to collect valid data in a community-based study, but more information is needed about their use among youth and their reliability for collecting longitudinal data. Adolescence is a developmental stage during which activity levels typically decline dramatically [7], and a call to action has been made to develop effective means of arresting this decline [8]. Most research studies include multiple time points of data collection, so it is critical to evaluate the validity of the data collected by these devices at multiple time points. Evidence for the validity and reliability of the Fitbit Zip as a tool that may be used in a supervised laboratory setting can now be considered fairly strong. However, initial forays into documenting the validity of the Fitbit Zip among free-living adults, where data quality can be impacted by undetected device malfunctions, non-wearing of the device, or issues with data loss or transfer, has revealed a number of factors that may limit the utility of this device outside the laboratory (e.g., potential for loss, battery failure, data uploading issues, etc.… [9]). Because youth engage in different patterns of physical activity as compared to adults [10], it is important that activity monitors be validated among youth as well as among adults. Obtaining valid measures of youth physical activity is necessary to evaluate behavior change interventions and investigate the link between physical activity and health among youth [11]. The purpose of this study was to investigate both the feasibility and the validity of using the Fitbit Zip to assess physical activity among free-living adolescents.

## Methods

### Participant recruitment

The study was conducted in three cohorts. The first cohort [N = 25; mean (SD) age = 12.76 (.72)] provided data in February and March of 2015, the second cohort [N = 35; mean (SD) age = 11.15 (.43)] provided data in September and October of 2015, and the third cohort [N = 27; mean (SD) age = 12.74 (.52)] provided data in February and March of 2016. The study was approved by the Institutional Review Board of the University of California, Irvine and the Long Beach Unified School District Research Review Committee. Students were recruited from a public middle school in Southern California via flyers and oral presentations that were given during their physical education (PE) class. Students and parents/guardians provided written informed assent and consent prior to participation. In Cohorts One and Three, students were recruited without regard to level of participation in physical activity. In Cohort Two, students were required to be a member of a sports team to participate in the study. All study participants were required to be eligible to participate in regular physical education, thus ensuring that they were generally healthy. The data collected for the present study were obtained as part of two larger ongoing studies. The inclusion criteria related to activity levels were imposed by the study protocols.

### Procedures

Each study participant underwent an assessment of height, weight and cardiorespiratory fitness (conducted at the school in a converted classroom) followed by 1 week of activity monitoring with the Fitbit Zip (San Francisco, CA, USA) attached to the belt of the ActiGraph activity monitor (model GT3X, ActiGraph, Pensacola, FL, USA). Prior to sending participants into the field with the activity monitors, the ActiGraph was fully charged and a new battery was installed in the Fitbit. Participants were instructed to wear the belt with both activity monitoring devices every day for 7 days, except when sleeping or bathing. Consistent with recommendations for obtaining a valid estimate of daily activity [12], 4 days of valid data was the minimum required to be considered complete. If a student returned incomplete data, as determined by the ActiGraph, the data collection was repeated. Fitbit accounts were established for each device by the research team. Students did not have access to their own accounts and were not given the password to view their data on-line.

### Measures

#### ActiGraph

The ActiGraph activity monitor is a tri-axial accelerometer that is attached to a belt that wraps around the hip and is not waterproof. It is marketed exclusively as a research device, and the cost of the ActiGraph is approximately $225. The ActiGraph is widely used in physical activity research and has been validated against objective measures of motion and of energy expenditure [13, 14]. The ActiGraph can store data for up to 40 days, is rechargeable, can run on battery power for up to 30 days, and syncs to a computer through a cable. A newer version of the device, not used in this study, can sync using Bluetooth.

#### Fitbit Zip

The Fitbit Zip, a tri-axial accelerometer, is marketed as a consumer-oriented device. The device is held by a silicon clip that can be attached essentially anywhere on the body, and is water-resistant. The Fitbit Zip can store data up to 7 days, and syncs wirelessly and automatically up to a 20-foot range. The cost of a Fitbit Zip is approximately $60 U.S., and it requires a non-rechargeable replaceable battery every 6 months. The Fitbit Zips used in this study were purchased new directly from the company in the fall of 2014.

#### Demographics

Students self-reported their age and ethnicity. Two questions determined ethnicity according to the format used by the National Institutes of Health. Students were first asked to indicate if they were Latino/Hispanic (yes/no) and then asked to check a category indicating race (American Indian, Asian, African-American, Hawaiian/Pacific Islander, White, Multiracial, Other).

### Data analysis

Data from the ActiGraph were aggregated using the Actilife software with the following parameters specified: (1) a valid day included at least 8 h of wear time; (2) for an hour to be included in wear time, it could not include a string of 30 min with zero activity; (3) data must be available for a minimum of 4 valid days. The Actilife software was used to yield both mean daily minutes of MVPA and mean daily steps across all valid days. The threshold for activity to be included as MVPA was computed using the formula recommended by Freedson [15] for children and using 4 METS (metabolic equivalents) as the minimum threshold for MVPA. For Cohorts One and Three, the average age of participants was 12 years, and the cutoff for MVPA yielded by the Freedson equation was 2058 counts per minute. Cohort Two participants were slightly younger (average age was 11 years old) so the cutoff for MVPA yielded by the equation was 2220 counts per minute. These parameters are easily specified in the software, and can be used to analyze all participant data simultaneously to yield a number that represents average daily minutes-per-day of MVPA. The selection of the relatively stringent criterion for non-wear time (i.e., a string of 30 min with zero activity) has been criticized in other studies for the potential to create a biased sample [16]. However, since we repeated the assessment until all participants met the inclusion criterion, no participants were excluded on the basis of failing to meet the criterion for valid wear time.

Utilizing the Fitabase software (Small Steps Lab, San Diego, CA, USA), data from the Fitbit Zip were exported as minute-by-minute data. The minute-by-minute data were used to verify that each participant included in the analyses had at least 4 valid days of data, with a valid day being defined as at least 8 h of valid data (a valid hour being one that did not contain more than 20 consecutive minutes of zero steps). Including only valid hours, the minute-by-minute data then were used to compute mean daily steps and MVPA (i.e., the total of what Fitbit calls “active” and “very active” minutes) for all valid days. Fitbit does not provide access to the raw counts-per-minute data. We explored using a string of 30 consecutive zero steps minutes as the criterion for a valid hour, to mirror the approach used with the ActiGraph, but found that this criterion was overly strict and resulted in very few valid hours on the Fitbit data. Using 20 consecutive zero steps minutes effectively eliminated obvious non-wear time and avoided the exclusion of periods during which the participant may have been minimally active but still wearing the device.

Comparisons of the average minutes-per-day of MVPA and average steps per day derived by the two instruments were conducted using Pearson’s correlations and Bland–Altman analyses. To examine whether there was a systematic difference between the devices, a one-sample t test was conducted to compare the mean difference between the estimates from the two devices (e.g., ActiGraph steps—Fitbit steps) and zero. A regression analysis in which the difference between the two device estimates was regressed on the mean of the two estimates was used to expose any proportional bias (i.e., change in the correlation between the two devices in relation to the magnitude of activity). In constructing the Bland–Altman plot, the y-axis represents the difference between the ActiGraph and the Fitbit estimates, and the x-axis represents the mean of the two estimates [17].

## Results

### Descriptive statistics

Table 1 shows the similarities and differences across the three samples. The samples in Cohorts One and Three were slightly older than the Cohort Two sample, but the gender and ethnic distributions were similar. As expected, given that participants in Cohort Two were required to be active in sports, the second cohort had higher cardiorespiratory fitness and was more active than the first and third cohorts, according to the ActiGraph assessments. Body mass index did not differ significantly between the groups.

**Table 1:** Participant characteristics and accelerometer data

	Cohort 1 (N = 25)	Cohort 2 (N = 35)	Cohort 3 (N = 27)
	%	%	%
Gender (% male)	48	47	40
Hispanic	32	44	41
Non-hispanic white	24	27	30
African–American	32	15	0
Multi-racial/other	12	12	22

	Mean (SD)	Mean (SD)	Mean (SD)
Age	12.76 (.72)	11.15 (.43)	12.74 (.52)
BMI percentile	66.88 (32.46)	59.17 (33.27)	62.29 (23.77)
VO2 peak (ml/kg/min)	34.95 (9.19)	44.00 (8.01)	37.69 (7.66)
Number of days of valid ActiGraph data	6.00 (.85)	5.49 (.83)	6.00 (.89)
Number of days of valid Fitbit data	6.04 (.97)	6.65 (.70)	6.10 (.89)
MVPA ActiGraph (min/day)	32.44 (12.91)	55.94 (26.89)	42.78 (26.14)
MVPA Fitbit (min/day)	117.63 (21.37)	52.84 (32.79)	40.07 (30.95)
Steps ActiGraph (per day)	7868 (1703)	10,271 (2504)	8523 (2866)
Steps Fitbit (per day)	8874 (1646)	10,723 (2853)	8921 (2911)

	*r*	*r*	*r*
MVPA correlation	.67	.79	.94
Steps correlation	.72	.92	.96

*p* for all *r*’s < .001

### Cohort One

**Fig. 1:**
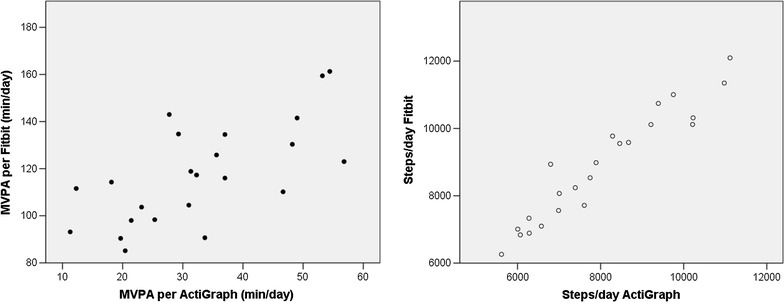
Correlations between the activity estimates from the Fitbit and the ActiGraph for Cohort One

Two of the Fitbits did not yield 4 days of valid data in Cohort One, so the sample size for the correlation analyses was 23. Correlation analysis indicated good correlation between the Fitbit and the ActiGraph (see Table 1; Fig. 1). Average daily MVPA assessed using the Fitbit was significantly and positively correlated with average daily MVPA assessed using the ActiGraph (r = .67, p < .001), and the correlation between steps-per-day assessed with the two devices was slightly stronger (r = .72, p < .001). However, there was a significant difference comparing the mean difference between the two assessments to zero for both MVPA (t = −25.79, p < .001) and steps (t = −8.32, p < .001), suggesting that the Fitbit overestimated activity as compared to the ActiGraph.

**Fig. 2:**
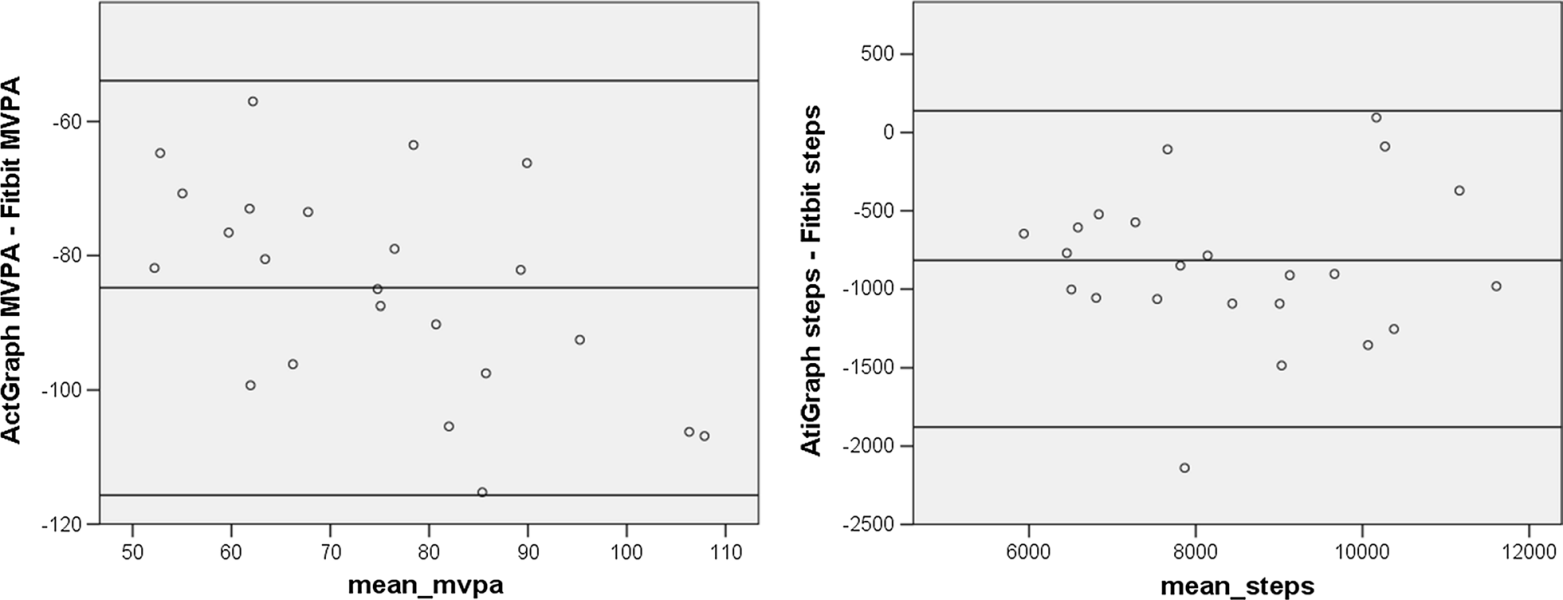
Bland–Altman plots for Cohort One. The *x-axis* represents the mean of the ActiGraph and Fitbit values. The *y-axis* represents the mathematical difference between ActiGraph values and Fitbit values

Figure 2 shows the Bland–Altman plots of the difference between the ActiGraph and the Fitbit estimates plotted against the mean of the two assessments. A regression of the difference between the ActiGraph and Fitbit estimates of MVPA on the mean of the two estimates was significant (t = −3.02, p < .01), indicating significant proportional bias, meaning that the degree to which the Fitbit overestimated MVPA in comparison to the ActiGraph increased as activity levels increased. A regression of the difference between ActiGraph and Fitibit estimates of steps per day on the mean of the two estimates was not significant, suggesting no proportional bias. However, the Fitbit overestimated steps by about 850 steps per day as compared to the ActiGraph.

### Cohort Two

**Fig. 3:**
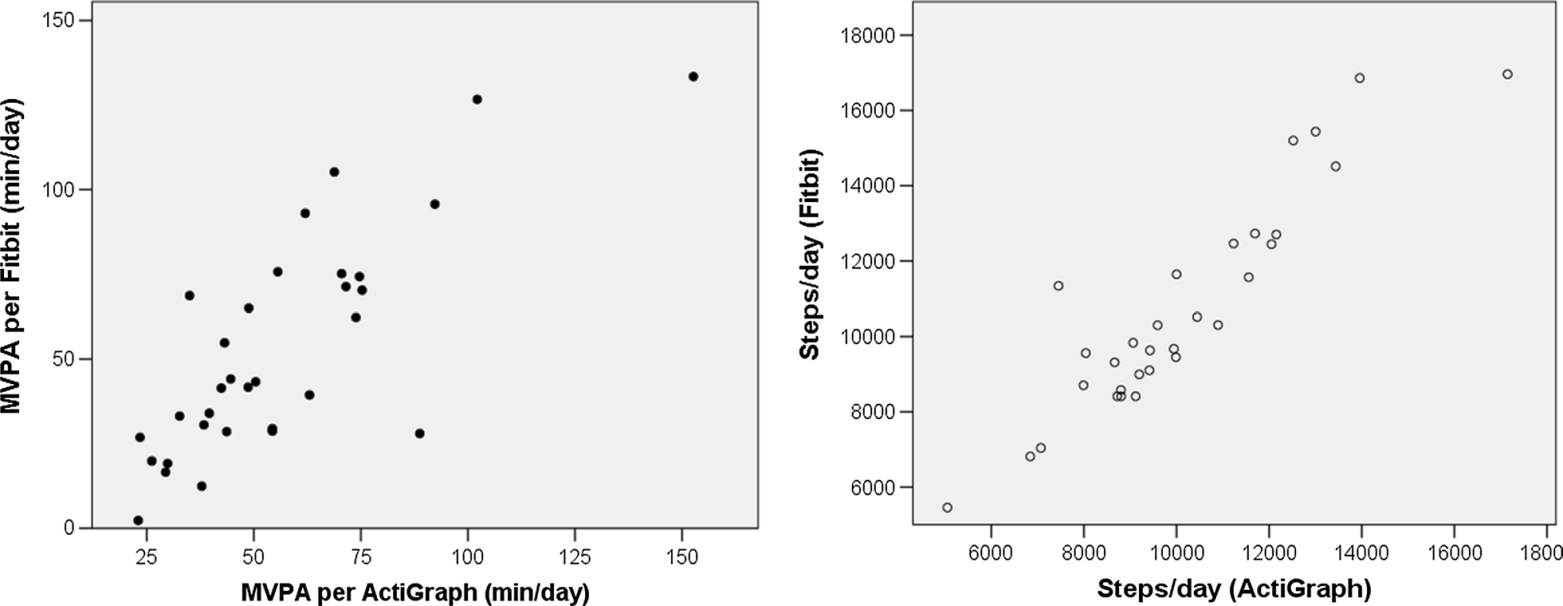
Correlations between the activity estimates from the Fitbit and the ActiGraph for Cohort Two

Screening of the Fitbit data from Cohort Two revealed that 5 devices failed to meet the criteria for 4 valid days of data, yielding a usable sample of 30 participants for the correlation analyses. Figure 3 illustrates the correlations. The correlation between the Fitbit and ActiGraph assessments of MVPA was strong (r = .79, p < .001). The correlation between the step counts for the two devices was even stronger (r = .92, p < .001).

**Fig. 4:**
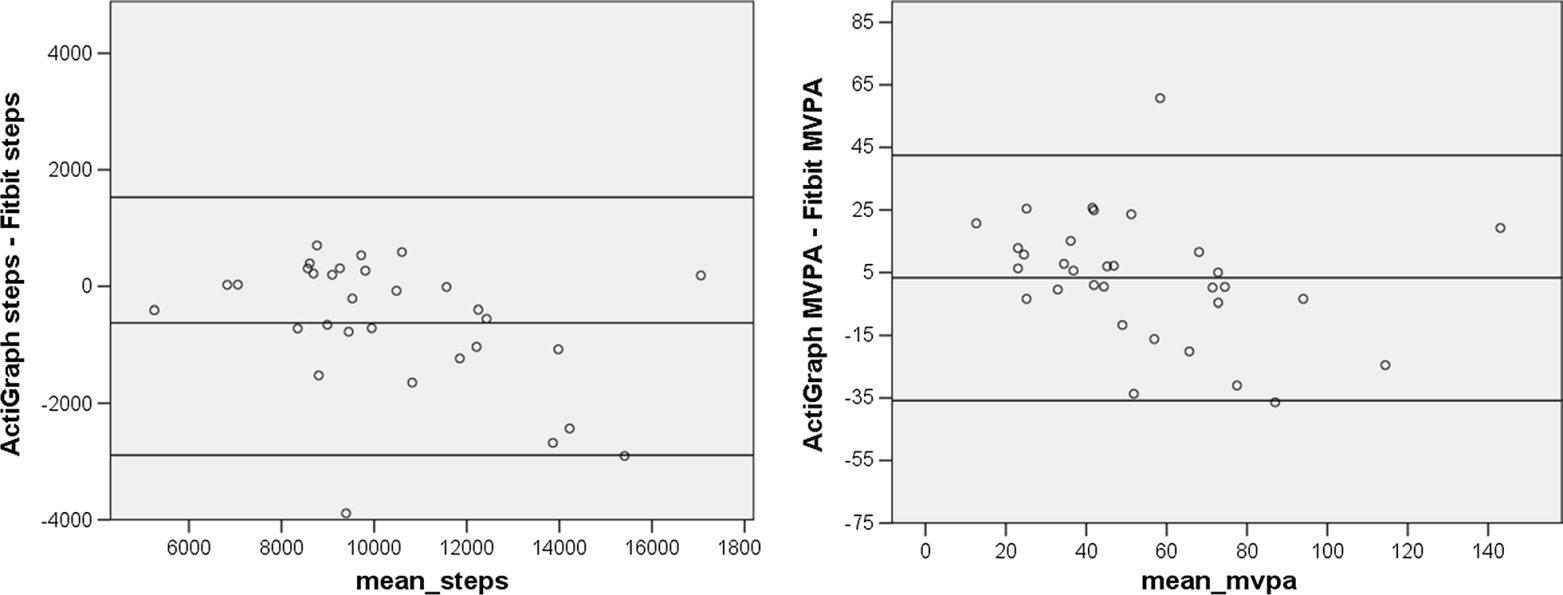
Bland–Altman plots for Cohort Two. The *x-axis* represents the mean of the ActiGraph and Fitbit values. The *y-axis* represents the mathematical difference between ActiGraph values and Fitbit values

Figure 4 examines the correlation between the two devices using Bland–Altman plots. In comparing the two devices for estimates of daily MVPA, a one-group t-test comparing the mean difference between the two assessments to zero revealed no significant difference (suggesting good agreement), a regression of the difference between the two assessments on the mean of the two assessments was not significant (indicating no proportional bias) and the magnitude of the mean difference between the two devices was less than 5 min per day, which is not clinically significant. Comparing the estimates of daily steps for the two devices, the t test of the differences between the two devices was significant (t = −3.06, p < .01; suggesting that the Fitbit overestimated steps in comparison to the ActiGraph), a regression of the difference between the two assessments on the mean of the two assessments was significant (t = −2.24, p < .05; suggesting a proportional bias, with overestimation increasing as activity increased), and the magnitude of the mean difference between the two devices was 617 steps per day.

### Cohort Three

**Fig. 5:**
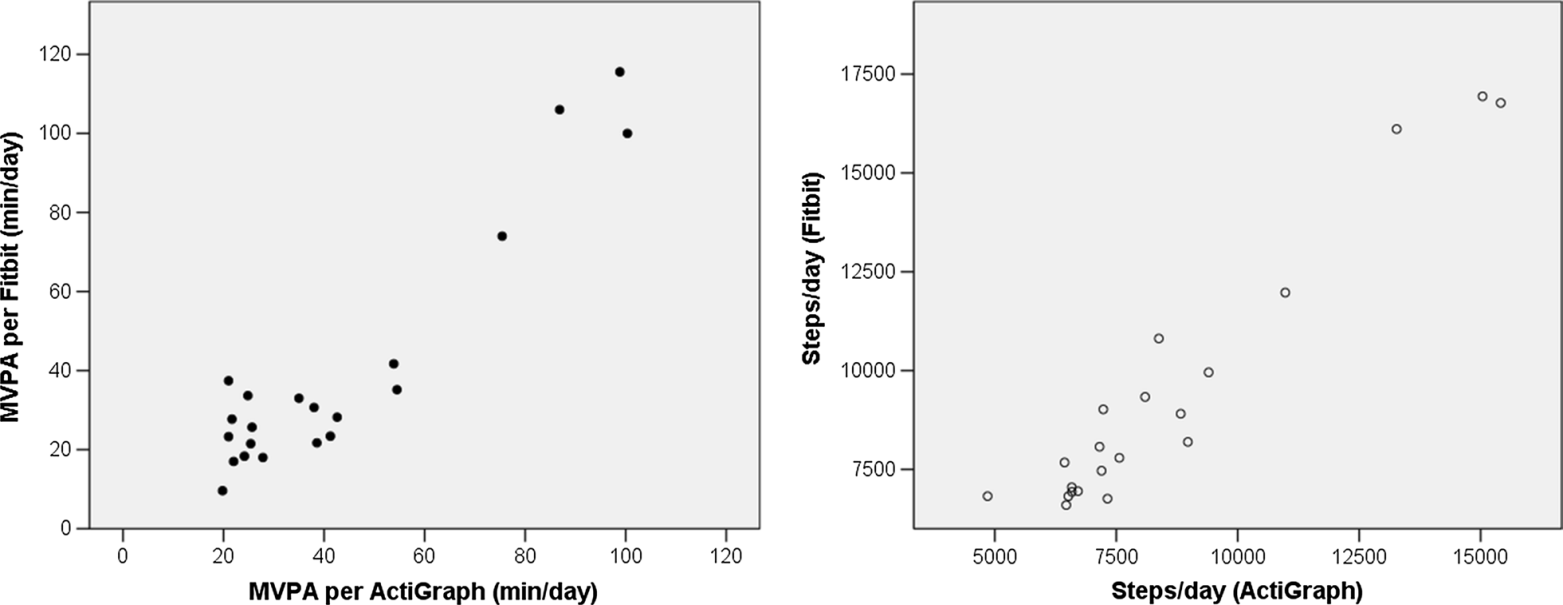
Correlations between the activity estimates from the Fitbit and the ActiGraph for Cohort Three

**Fig. 6:**
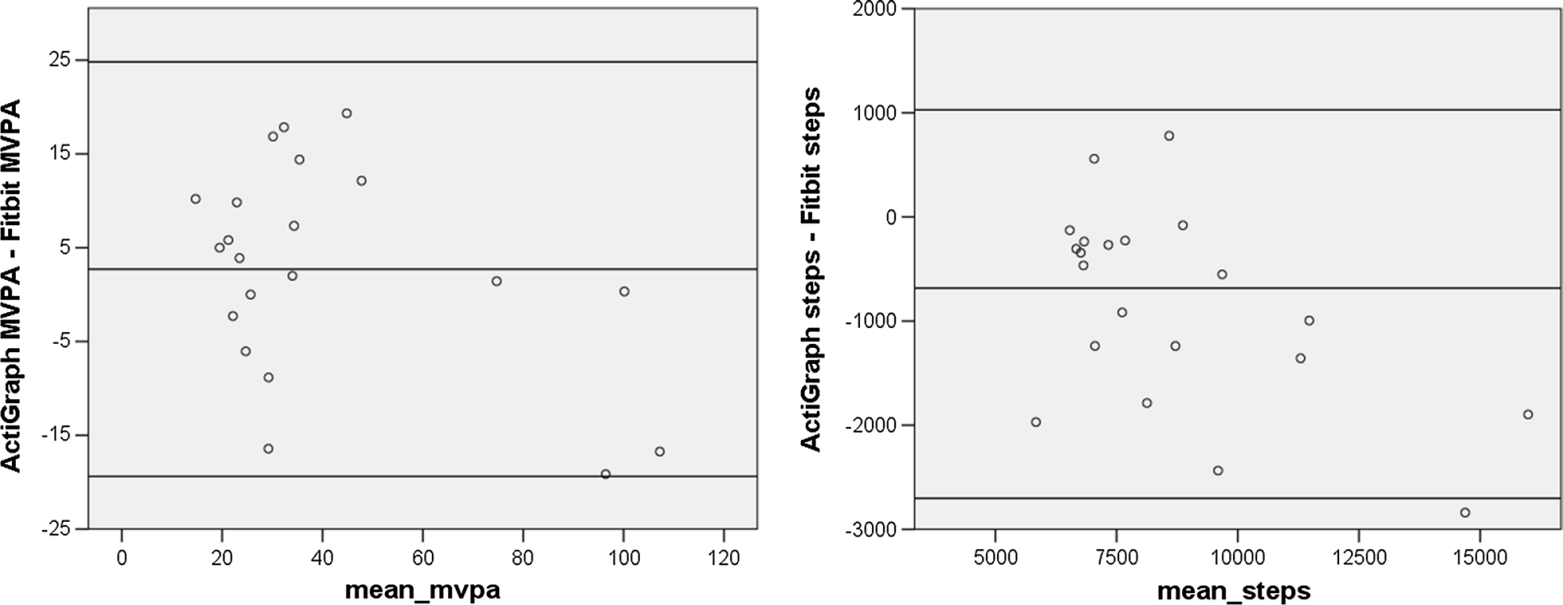
Bland–Altman plots for Cohort Three. The *x-axis* represents the mean of the ActiGraph and Fitbit values. The *y-axis* represents the mathematical difference between ActiGraph values and Fitbit values

In Cohort Three, data from six participants did not meet the criteria for inclusion, leaving 21 participants for analyses. Among these adolescents, and as illustrated in Fig. 5, the correlation between MVPA assessed with the ActiGraph and MVPA assessed with the Fitbit was very high (r = .94, p < .001), as was the correlation of step counts across the two devices (r = .96, p < .001). Figure 6 illustrates the Bland-Altman plots for Cohort Three. A comparison of the mean difference between the two devices to zero was not significant for MVPA, but it was for steps (t = −4.13, p < .01; suggesting overestimation by the Fitbit). A regression of the difference between the MVPA estimates on the mean for the MVPA estimates was not significant, suggesting that there was no proportional bias, and the magnitude of the difference between the two estimates was relatively small (mean difference was about 3 min per day). However, the regression of mean steps on the difference in steps was significant (t = −2.79, p < .05), indicating that the overestimation by the Fibit increased as activity levels increased, and the mean difference was 854 steps per day.

## Discussion

The proliferation of consumer-friendly activity monitoring devices has made physical activity research more accessible to investigators, but validation data in the field have been lacking, particularly with respect to using these devices among youth. This study set out to validate the use of the Fitbit Zip as a means of assessing physical activity levels among free-living adolescents. The results provide strong evidence of validity, yet also confirm concerns about potential changes in algorithms over time. Specifically, the Fibit correlated quite strongly with the ActiGraph among all three Cohorts for both estimates of steps per day and also estimates of daily MVPA. However, the Fitbit tended to overestimate steps overall, and overestimated MVPA by a considerable amount in Cohort One. Because this shift over time was so dramatic, we suspected it might be related to a change in the way that Fitbit processes the data, and in fact it emerged that Fibit modified their algorithms in April, 2015 (in between our Cohort 1 and Cohort 2 data collections) to include only activity that persisted for at least 10 min in their computation of “active minutes” (see https://www.help.fitbit.com/articles/en_US/Help_article/1379 for details). This change explains why the correspondence between the estimates of MVPA derived from the two devices was so much better for Cohorts Two and Three.

The Fibit’s overestimation of steps per day in comparison to the ActiGraph is consistent with the findings of Tully et al. [5]. These investigators found that the Fitbit overestimated steps by about 700 steps per day, which is a difference of a similar magnitude to what we found. Because the algorithms used to convert raw Fitbit data into indexes of activity (MVPA and steps) are not publicly available, it must be assumed that the company has chosen a threshold for steps that is lower than that used by the ActiGraph algorithm. There is vigorous debate and no generally-accepted standard for which algorithm is the most valid [18], so the mere fact that a different algorithm is used is not grounds for rejecting the Fitbit. It is important, however, to be cognizant that activity metrics obtained from the Fitbit should probably not be treated as equivalent to activity metrics obtained from the ActiGraph. However, the MVPA data obtained from Cohorts Two and Three in this study showed very close correspondence between the Fitbit and the ActiGraph (i.e., very high correlations and no statistical difference between the means), suggesting that the Fitbit might in fact offer a less expensive alternative to the ActiGraph.

It has been argued that consumer-oriented devices such as the Fitbit Zip may offer a valuable adjunct to behavior-change interventions by providing accessible feedback regarding activity levels, but should not replace research-grade devices in collecting data to be used in scientific analyses [19]. A strong objection to using consumer-oriented devices to collect research data is that the companies guard their algorithms closely, which leaves scientists in the dark as to whether the algorithms have been changed over time and how the algorithms relate to those of known devices, such as the ActiGraph. The present study gives credence to this concern, and should raise alarms about the possibility that a device company might alter their algorithms in the middle of a longitudinal research study.

Clinicians also are eager to have access to an accurate and reliable device to track activity among their patients, and lab-based studies have been presented as evidence for using the Fitbit in a clinical setting [20]. However, as this study demonstrates, accuracy in a controlled laboratory setting may not translate to validity and reliability over time. Our results suggest that both clinical and research utilization of consumer-oriented devices to measure and assess physical activity should proceed cautiously and that additional evidence of field-based performance is critical.

Another point that should be made is that these adolescents were wearing an ActiGraph on a belt throughout the week of data collection. The Fitbit was affixed to the ActiGraph belt. Consequently, there was no need for the study participant to keep track of the Fitbit. It has been our general experience working with this device, which clips onto a pocket or waistband, that individuals do not infrequently forget either to remove the device from their clothing at the end of the day (at times resulting in the device making a journey through a washing machine) or to attach the device to their clothing in the morning, resulting in data loss. Because the ActiGraph belt is more difficult to overlook, participants generally have an easier time remembering to remove it and replace it as instructed. Recently, Fitbit has released a line of wrist-worn devices that employ similar technology for slightly greater expense. Because these wrist-worn devices are less intrusive and can be worn 24 h per day, they may solve this issue of wearability. Studies are beginning to emerge validating the wrist-worn monitors [4], and it is likely that if they prove valid and reliable they will be seen as preferable to the waist-worn devices.

Practically speaking, this study suggests that the Fitbit Zip is a reliable assessment tool among adolescents when worn for at least 4 days and when used within a period of time during which Fitbit does not modify their algorithms. Because it is far more affordable than the ActiGraph, the Fitbit Zip may therefore be a feasible tool for assessing activity levels across large groups of youth, perhaps within a school setting, as a means of tracking group changes in activity over time.

## Conclusions

Several studies have previously demonstrated the validity of the Fitbit in a controlled laboratory setting. This study takes the analysis out into the field and evaluates the device among free-living adolescents. The results offer confirmation of a high correlation between Fitbit and ActiGraph data for both MVPA and steps per day, although steps per day tend to be overestimated by the Fitbit in comparison to the ActiGraph. It should also be noted that a large part of the appeal of the Fitbit is the potential for using it over longer periods of time than a 7-day assessment. This study does not provide information about the utility of the Fitbit for collecting data over periods longer than 1 week.
